# Identification of unknown colorants in pre-Columbian textiles dyed with American cochineal (*Dactylopius coccus* Costa) using high-performance liquid chromatography and tandem mass spectrometry

**DOI:** 10.1007/s00216-014-8107-y

**Published:** 2014-09-12

**Authors:** Katarzyna Lech, Katarzyna Witkoś, Beata Wileńska, Maciej Jarosz

**Affiliations:** 1 Faculty of Chemistry, Chair of Analytical Chemistry, Warsaw University of Technology, Noakowskiego 3, 00-664 Warsaw, Poland; 2 Faculty of Chemistry, University of Warsaw, Pasteura 1, 02-093 Warsaw, Poland

**Keywords:** American cochineal, Natural dyes, Pre-Columbian Peruvian textiles, HPLC, Tandem mass spectrometry

## Abstract

The present study concerns the identification of nine thus-far unknown derivatives of carminic acid extracted from pre-Columbian Peruvian textiles dyed with American cochineal—these derivatives are not found in commercially available preparations of the dye. These compounds probably represent a unique fingerprint of dyed textiles from this region, as they have never been reported to occur in other fabrics of historical value. They were separated by reversed-phase high-performance liquid chromatography (phenyl column) and detected using a UV/vis spectrophotometer and two tandem mass spectrometers. Peaks observed in chromatograms registered at 450 and 500 nm were further identified by ESI QqQ MS (mainly in the negative ion mode), supported by high-resolution ESI QIT/ToF MS data. The characteristic fragmentation pathways of isolated carminic acid and its derivatives provided additional information concerning lost neutrals and thus the functional groups and substituents present in the parent molecules. This information mainly related to multiple cleavages of the hexoside moiety (initially cross-ring cleavage), which are characteristic of *C*-glucosides (loss of 90, 120, and 148 Da). This is accompanied by the elimination of H_2_O as well as the further loss of 60 Da from the hexoside moiety. Moreover, other losses from the carbonyl groups (44 Da from CO_2_ loss, 62 Da from ethylene glycol loss, 32 Da from O_2_ loss, 138 Da from hydroxybenzoic acid, and 120 Da from oxomethylene cyclohexadienone) provided more specific information about structures of the identified derivatives of carminic acid.

## Introduction

Pre-Columbian cultures developed in South America until they were conquered or significantly influenced by Europeans. For instance, Ancient Peru was inhabited for thousands of years by successive great indigenous civilizations, such as the Chuquibamba, Chimú, Chancay, and Inca cultures. In the fifteenth century, most of the contemporary cultures in the region joined with the Incas, who created the largest empire in pre-Columbian America. This empire was conquered in 1532 by the Spanish. The most common works of art left behind by all of these cultures were textiles, and the dye that gives them their dominant red-pink shade derives from an insect called the cochineal (*Dactylopius coccus* Costa). This insect is one of nine species found in the genus *Dactylopius*, which are native to tropical and subtropical South America and Mexico [1]. It was not used by the initial and early pre-Columbian cultures before the third century BC [1]. Cochineal dye began to appear more frequently in ancient Peru in the Huari and Tiahuanaco period (700–1100 AD) [1, 2]. It is currently the most popular natural red dye, and it owes its quality to its high content of coloring compounds, mainly carminic acid (94–98 %). Minor constituents of cochineal dye (i.e., the remaining 2-6 %) include kermesic and flavokermesic acid, dcII, dcIV, and dcVII [3].

Textile is a unique and difficult material to study. On the one hand, ancient textiles are of great artistic and historical value, which often limits the potential availability of a sample of a textile of interest; on the other hand, such textiles usually contain many colorants (often present at trace levels) which are a rich source of knowledge, but this knowledge is only obtained when the colorants are analyzed in an appropriate way. For these reasons, identifying the dyes in historical objects requires the use of sensitive and selective analytical techniques. High-performance liquid chromatography coupled with spectrophotometric and mass-spectrometric detectors (HPLC–UV-Vis–MS) has proven to be a useful tool for analyzing works of art, especially those containing organic compounds such as natural colorants [4, 5]. Electrospray ionization in the negative ion mode permits the analysis of polyphenolic glycosides, while tandem mass spectrometry (MS/MS) enables the identification of such compounds, even those of unknown structure. Collision-induced dissociation (CID) leads to the formation of Y_*j*_ ions of *O*-glycosides (glycosidic cleavages) and ^*k*,*l*^X_*j*_ ions of *C*-glycosides (cross-ring cleavages), where the superscripts *k* and *l* indicate cleavage links in carbohydrate rings and the subscript *j* refers to the number of interglycosidic bonds [6, 7]. However, fragmentation may also occur through the scission of other bonds and/or the loss of small neutral molecules such as H_2_O (18 Da), CO (28 Da), CH_2_O (30 Da), and CO_2_ (44 Da) [6, 8–10].

Color components of American cochineal have been examined in several studies. One of the first investigations helped to define the structure of flavokermesic acid by confirming its identity with laccaic acid D [11]. Subsequent studies, carried out mainly using HPLC, have been devoted to identifying minor components of the dyestuff: dcII, dcIV, and dcVII [9, 12]. Their presence in analyzed textiles (pre-Columbian [13, 14] as well as Renaissance and later European ones [9, 15–17]) provides the basis for identifying American cochineal. The studies were carried out using HPLC–UV-vis [13, 18] and HPLC–UV-vis–MS [9, 14–16, 19]. However, the structures of the minor colorants observed in American cochineal have been unambiguously identified only relatively recently [17]; information obtained from nuclear magnetic resonance (NMR) experiments has shown that dcII is 7-*C*-α-D-glucopyranoside of flavokermesic acid, dcIV is 7-*C*-α-D-glucofuranoside of kermesic acid, and dcVII is 7-*C*-β-D-glucofuranoside. Additionally, three further compounds have been also found and identified in American cochineal: dcIII is 7-*C*-α-D-glucopyranoside of 5-aminokermesic acid, dcOfka is 6-*O*-α-D-glucopyranoside of flavokermesic acid, and ddca is 5,6-dideoxycarminic acid.

In the present study, HPLC–UV-vis–ESI QqQ MS and HPLC–ESI QIT/ToF MS were used to achieve the standardless identification of nine thus-far unknown coloring compounds (dc1–dc9) extracted from fibers taken from pre-Columbian textiles dyed red with American cochineal. These colorants, observed using a spectrophotometric detector at 450 and 500 nm, were identified based on MS/MS spectra registered in the negative ion mode.

## Experimental

### Apparatus

Separation and identification of the colorants were carried out using a liquid chromatographic system with spectrophotometric and two tandem mass-spectrometric detectors, ESI QqQ MS and ESI QIT/ToF MS. The optimized parameters of the developed method are presented in Table 1. The samples were injected onto a column using an injection valve, a model 7225i from Rheodyne (Cotati, CA, USA). The mobile phase was degassed using a model 1100 micro vacuum degasser (Agilent Technologies, Santa Clara, CA, USA), and the analyses were controlled by the MassHunter Workstation software (Agilent Technologies) or LCMS solutions software (Schimadzu Corporation, Kyoto, Japan).

**Table 1:** Conditions employed for HPLC–UV-vis–ESI MS separation and detection of carminic acid derivatives

HPLC separation			
Pump	LC 1100 quaternary pump (Agilent Technologies)		
Column	Zorbax SB-Phenyl, 4.6 × 150 mm, 3.5 μm, 80 Å (Agilent Technologies)		
Precolumn	Zorbax SB-Phenyl, 4.6 × 12.5 mm, 5.0 μm (Agilent Technologies)		
Injection volume	20 μL (Rheodyne model 7225i)		
Flow rate	0.5 mL min^−1^		
Eluents	(A) 1.5 % (v/v) formic acid in water, (B) methanol		
Gradient program	time, min	% A	% B
	0.0	60	40
	15.0	40	60
	20.0	30	70
	27.0	0	100
	30.0	0	100
Stop time	30.0 min		
Equilibrium time	8.0 min		
UV-vis detection			
Detector	VWD 1200 (Agilent Technologies)		
Wavelength	280, 450, 500, 550 nm		
ESI-MS detection			
Detector	6460 triple quad LC/MS with JetStream Technology (Agilent Technologies)	LCMS-IT-TOF (Shimadzu Corporation)	
Polarity	Negative (NI)	Negative (NI)	
Mode	Scan, product ion	Scan, product ion	
Mass range	*m*/*z* 100–800	*m*/*z* 100–800	
Ionization/Interface voltage	3000 V	1800 V	
Orifice voltage	100 V	–	
Drying gas flow	6 L min^−1^	–	
Drying gas temperature	300 °C	–	
Nebulizer pressure	45 psi	–	
Nebulizer gas flow	–	1.5 L min^−1^	
Sheath gas flow	12 L min^−1^	–	
Sheath gas temperature	380 °C	–	
Nozzle voltage	500 V	–	
CDL temperature	–	250 °C	
Heat block temperature	–	250 °C	
Collision energy	25 or 35 V (NI)	30 or 50 %	
Collision gas	–	30 or 50 %	

Extraction of the colorants from the fibers was performed using an ultrasonic bath (model 1210, Branson, Danbury, CT, USA), as well as with a water bath (WB 10, Memmert, Schwabach, Germany).

### Chemicals and materials

*Standards*: carminic acid of analytical chemical grade was purchased from Fluka (Buchs, Switzerland); kermesic acid was kindly donated by Dr. Ioannis Karapanagiotis (“Ormylia” Art Diagnosis Centre, Greece); flavokermesic acid was obtained from a mixture of natural products known as lac dye. Cochineal (*Dactylopius coccus* Costa) and lac dye were purchased from Kremer-Pigmente (Aichstetten, Germany).

Methanol of LC/MS purity was purchased from POCH (Gliwice, Poland) and analytical grade hydrochloric acid (35–38 %) was obtained from AppliChem (Darmstadt, Germany). Demineralized water was obtained from a Milli-Q Elix 3 system from Millipore (Molsheim, France).

Examined fibers were taken from two pre-Columbian textiles provided by Ewa Soszko from The Textile Conservation Department of Academy of Fine Art in Warsaw:

- Red thread from a plaid woollen fabric, in the middle of which is a belt presenting geometrically simplified images of animals. The textile dates from the Inca culture (1200–1532 AD) and belongs to the collection of the State Ethnographic Museum in Warsaw (inventory number 15885).
- Purple thread from a woollen tapestry depicting an eight-pointed star. Textile is from the Chuquibamba culture (1200–1450 AD), and is from a private collection (catalog number KPT8G).

### Standard solutions

Two milligrams of each standard preparation were dissolved in 10 mL of methanol. The obtained solutions were filtered over a 0.45-μm PET syringe filter (PPHU Q3 S.C., Brzeziny, Poland). The first five drops were discarded, and only the part of the filtrate that remained after dilution was used for the analysis.

### Extraction procedures

Twenty milligrams of ground dried cochineal were extracted with 10 mL of methanol. The solution was kept in an ultrasonic bath for 5 min, in a water bath (at 60 °C) for the next 15 min, and then filtered over a 0.45-μm PET syringe filter and analyzed as described above.

Fiber samples (0.2–0.3 mg) were extracted by adding them to 50 μL of a mixture of methanol and 37 % hydrochloric acid (17:3, v/v) and placing this mixture in an ultrasonic bath for 10 min and in a water bath (at 60 °C) for 25 min. The obtained extracts were separated from the fiber and diluted with 50 μL of water.

## Results and discussion

The aim of the study was to identify new coloring compounds present in extracts from pre-Columbian textiles dyed with cochineal. Before analyzing the historical threads, American cochineal was carefully examined by an HPLC–UV-vis–ESI QqQ MS system with a reverse-phase phenyl column. At first, MS detection was performed in the negative ion full-scan mode, which allowed deprotonated quasi-molecular ions ([M − H]^−^) to be selected for further MS/MS analysis in product ion and neutral loss modes using different CID energies. Finally, the identification of thus-far unknown compounds was confirmed by analyzing the high-resolution data from ESI QIT/ToF MS. The retention times of all separated compounds present in both cochineal and extracts from the fibers, the nominal and exact masses of their deprotonated quasi-molecular ions, as well as their proposed formulae are presented in Table 2.

**Table 2:** Anthraquinone compounds extracted from American cochineal and pre-Columbian fibers dyed with American cochineal

	*t*_R_ (min)	[M − H]^−^, *m*/*z*		Elemental composition	Diff (ppm)	Characteristic	Fragment ions, *m*/*z* (CE, V)
		Nominal	Highly resolved				
dc1	9.9	475	475.0892	C_22_H_19_O_12_	2.10	*C*-glucoside, isomer of dcII	431 (15), 341 (25), 311 (20), 282 (35)
dc2	10.0	521	521.0589	C_22_H_17_O_15_	3.07	*C*-glucoside, dicarboxylic acid	477 (10), 433 (20), 343 (25)
dcII	11.1	475	475.0883	C_22_H_19_O_12_	0.21	*C*-glucopyranoside of flavokermesic acid	431 (14), 341 (23), 311 (24), 282 (41)*
ca	11.9	491	491.0840	C_22_H_19_O_13_	1.83	carminic acid (*C*-glucopyranoside of kermesic acid)	447 (15), 357 (24), 327 (25)*
dcIII	14.3	490	490.0987	C_22_H_20_NO_12_	−0.82	*C*-glucopyranoside of 5-aminokermesic acid	446 (20), 356 (25), 326 (25)*
dc3	15.3	535	535.1092	C_24_H_23_O_14_	−0.19	*C*-glucoside, hydroxyethyl ester of carminic acid ((hydroxyethyl)carminic acid)	473 (20), 445 (30), 415 (25)
dcOfka	15.5	475	475.0881	C_22_H_19_O_12_	−0.21	*O*-glucopyranoside of flavokermesic acid	431 (18), 268 (35)*
dc4	16.4	519	519.0778	C_23_H_19_O_14_	−0.39	Isomer of dc6, derivative of carminic acid with an additional CO moiety (methyleneoxy group), probably carboperoxate	397 (30), 385 (25), 327 (35)
dc5	16.6	489	489.0677	C_22_H_17_O_13_	0.41	Dehydrocarminic acid, *C*-glucoside	487 (20), 399 (25), 369 (25)
dcIV	17.6	491	491.0836	C_22_H_19_O_13_	1.02	*C*-glucofuranoside of kermesic acid (isomer of carminic acid)	447 (15), 357 (24), 327 (26), 284 (32)*
dc6	18.0	519	519.0779	C_23_H_19_O_14_	−0.19	isomer of dc4, derivative of carminic acid with an additional CO moiety (methyleneoxy group), probably peroxide	487 (15), 399 (30)
dc7	18.8	611	611.1037	C_29_H_23_O_15_	−0.82	*C*-glucoside, carboxyphenyl ester of carminic acid ((carminyloxy)benzoic acid)	429 (20), 309 (35)
dc8	19.0	447	447.0942	C_21_H_19_O_11_	2.01	*C*-glucoside, decarboxycarminic acid	447 (15), 357 (24), 327 (25)
dcVII	19.5	491	491.0839	C_22_H_19_O_13_	1.63	*C*-glucofuranoside of kermesic acid (isomer of carminic acid)	357 (20), 327 (25), 299 (35)*
dc9	22.0	611	611.1075	C_29_H_23_O_15_	5.40	*C*-glucoside, carminic acid derivative with an additional –C_7_H_5_O_2_ moiety at the C-2′ position (carminic acid-2′-(4-hydroxybenzoate) or carminic acid-2′-salicylate)	567 (20), 429 (25), 257 (30), 327 (30)
fa	23.9	313	313.0354	C_16_H_9_O_7_	0.0	Flavokermesic acid	269 (5), 257 (17)*
ka	24.7	329	329.0301	C_16_H_9_O_8_	−0.61	Kermesic acid	285 (4), 257 (18)*

* Collision energy optimized using standard solutions or cochineal extract

### Cochineal revisited

#### Fragmentation paths of carminic acid and its isomers

**Fig. 1a–c:**
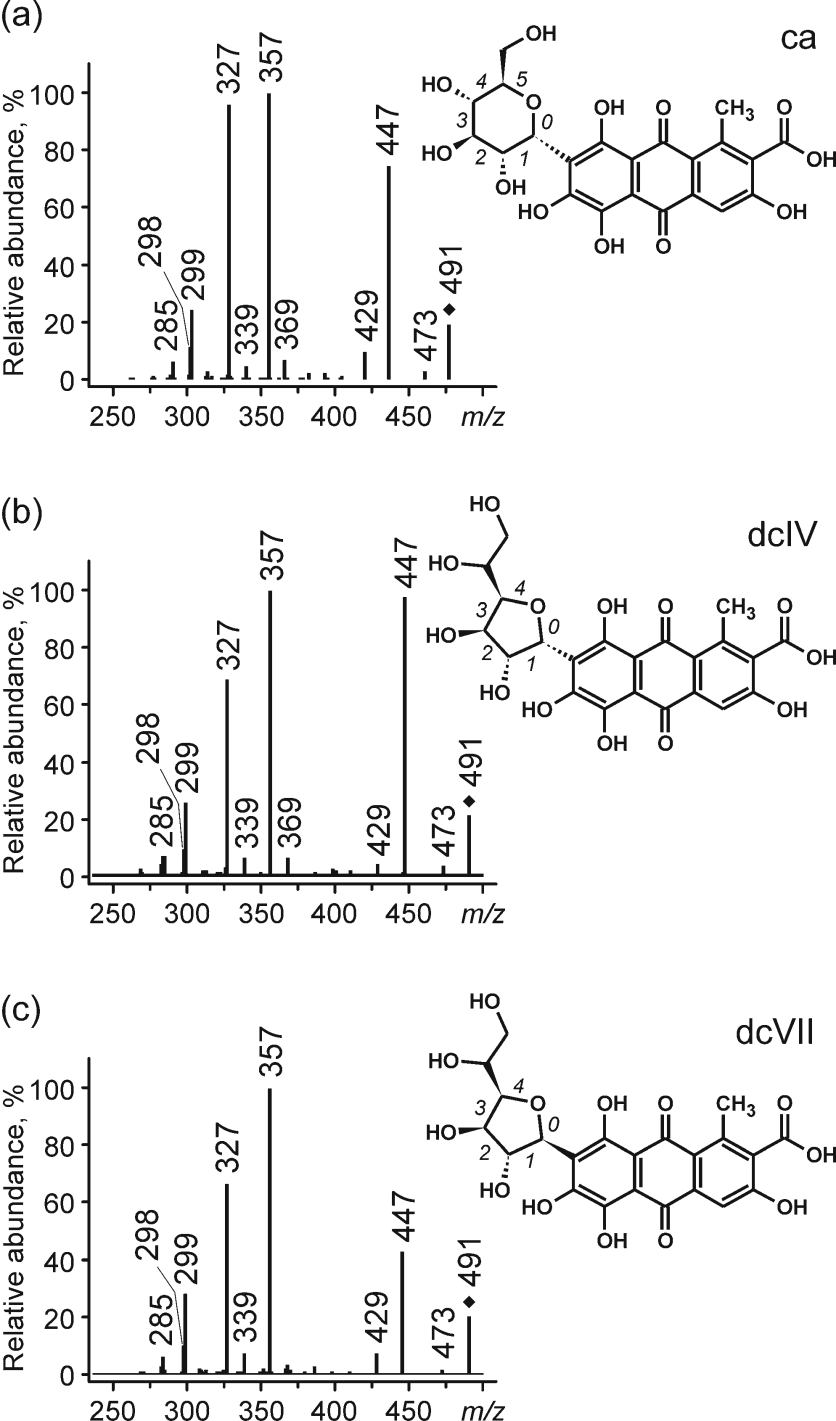
ESI QqQ MS product ion spectra of **a** carminic acid, **b** dcIV, and **c** dcVII (parent ion at *m*/*z* 491, CE 20 V)

**Scheme 1:**
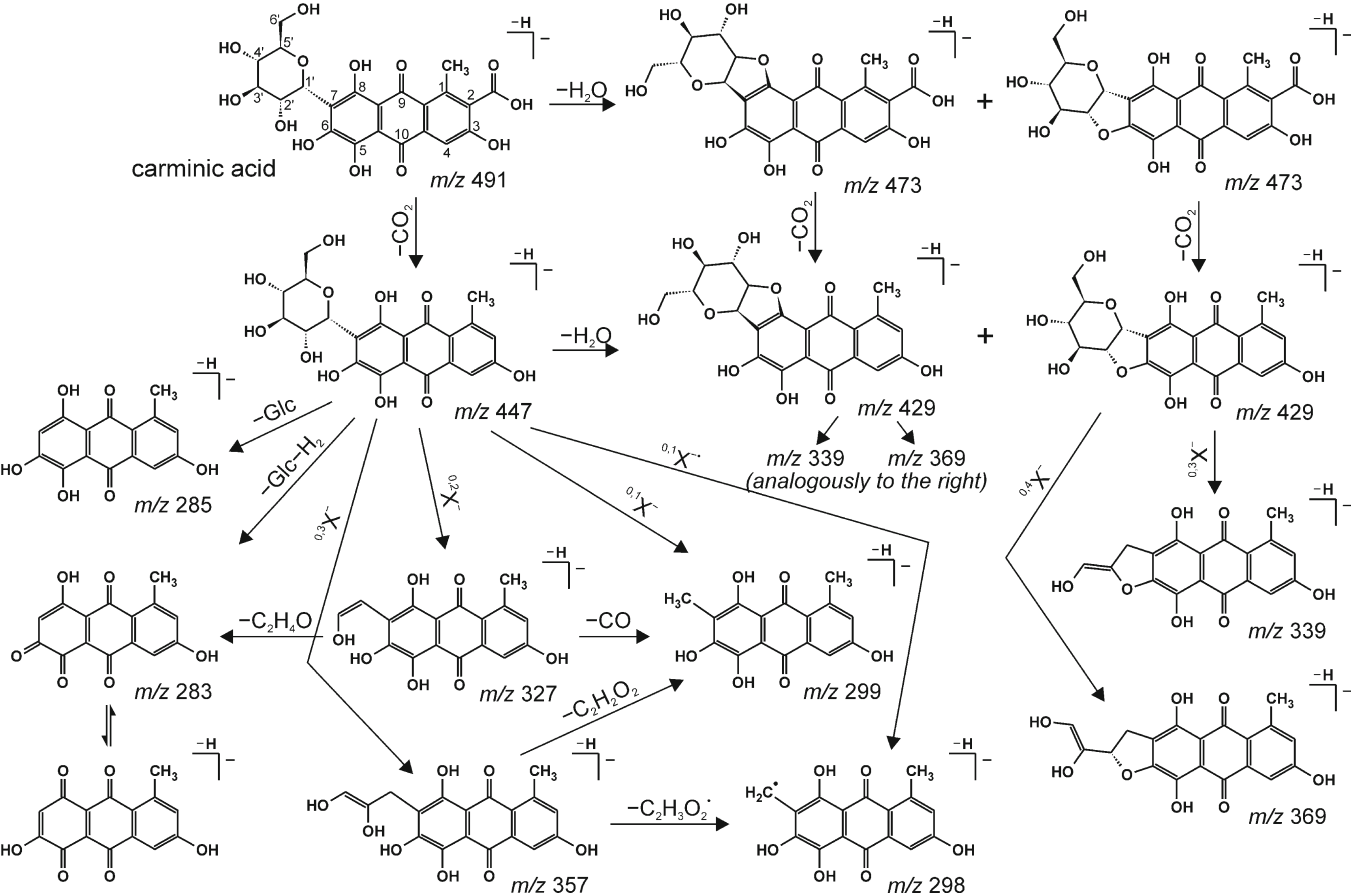
Postulated fragmentation pathways of carminic acid

Carminic acid, the main colorant of American cochineal, is 7-*C*-α-D-glucopyranoside of kermesic acid. DcIV (7-*C*-α-D-glucofuranoside of kermesic acid) and dcVII (7-*C*-β-D-glucofuranoside) [17], its isomers, differ from it only in a sugar moiety. Their MS/MS spectra obtained in the product ion mode (Fig. 1) are similar and the main observed losses are typical of carboxylic acids and *C*-glycosides. A relatively small collision energy (below a CE of 15 V) is sufficient to fragment the quasi-molecular ions (*m*/*z* 491) through the loss of CO_2_ (observed at *m*/*z* 447), but much richer spectra are obtained with a higher CE (e.g., 20 V); careful interpretation of those spectra allows us to propose two fragmentation paths of the examined derivatives of carminic acid, as presented in Scheme 1.

The first one is a series involving fragmentations of sugar moieties, typical of *C*-glucosides, following the loss of carbon dioxide. Consequently, the losses of characteristic neutrals of 90, 120, and 148 Da (C_3_H_6_O_3_, C_4_H_8_O_4_, and C_5_H_8_O_5_) result in ions at *m*/*z* 357 [^0,3^X − H − CO_2_]^−^, 327 [^0,2^X − H − CO_2_]^−^, 299 [^0,1^X − H − CO_2_]^−^, and 298 [^0,1^X − H − CO_2_]^−•^, respectively. In the spectra recorded only with collision energies >15 V, two other signals at *m*/*z* 285 and 283 can be observed, but their intensities are very small. They are formed by the heterolytic cleavage of the bond between the glucose moiety and aglycone (162-Da or 164-Da loss).

The ions at *m*/*z* 473, 429, 369, and 339 reflect a second fragmentation path involving the loss of a water molecule. This has been previously observed for some flavone *C*-glycosides [7, 20]. By analogy, elimination of water from carminic acid (or its isomers) can occur between the hydroxyl substituent at position C-2′ of the sugar moiety and the hydroxyl groups at the C-6 or C-8 position of the aglycone, which is also facilitated by hydrogen bonding between the ether oxygen atom of the sugar ring and the 6- or 8-hydroxyl group. This loss results in the formation of an [M − H − H_2_O]^−^ ion that registers at *m*/*z* 473 and [M − H − H_2_O − CO_2_]^−^ at *m*/*z* 429. Moreover, the formation of a dihydrofuran moiety between the sugar and aglycone parts leads to changes in the further fragmentation of the glycone ring. This is confirmed by the appearance of the signal at *m*/*z* 369 of the [^0,4^X − H − H_2_O − CO_2_]^−^ ion (characteristic loss of 60 Da), which is not a product of the classical decomposition of *C*-glucosides, as well as the presence of a peak at *m*/*z* 339 ([^0,3^X − H − H_2_O − CO_2_]^−^, loss of 90 Da). At the same time, there is no trace of 0–2 cleavage.

#### Other carboxylic acid colorants

DcII (7-*C*-α-D-glucopyranoside of flavokermesic acid) differs from carminic acid only in its lack of a hydroxyl group at the C-5 position (leading to a 16-Da lower molecular mass). Instead of this hydroxyl group, the molecule of dcIII (7-*C*-α-D-glucopyranoside of 5-aminokermesic acid) has a primary amine group (resulting in a 1-Da lower molecular mass). The fragmentation paths of their quasi-molecular ions (*m*/*z* 475 and 490, respectively) are almost identical to these discussed above for carminic acid, and a series of signals are observed: [M − H − H_2_O]^−^ (*m*/*z* 457 and 472), [M − H − CO_2_]^−^ (*m*/*z* 431 and 446), [M − H − H_2_O − CO_2_]^−^ (*m*/*z* 413 and 428), [^0,4^X − H − H_2_O − CO_2_]^−^ (*m*/*z* 353 and 368), [^0,3^X − H − CO_2_]^−^ (*m*/*z* 341 and 356), [^0,3^X − H − H_2_O − CO_2_]^−^ (*m*/*z* 323 and 338), [^0,2^X − H − CO_2_]^−^ (*m*/*z* 311 and 326), [^0,1^X − H − CO_2_]^−^ (*m*/*z* 283 and 298), [^0,1^X − H − CO_2_]^−•^ (*m*/*z* 282 and 297), and [M − H − Glc]^−^ (*m*/*z* 269 and 284).

However, in the spectrum of dcIII, the signals from ions formed by the loss of a water molecule is much more intense than those due to the loss of carbon dioxide (contrary to what is seen for carminic acid). This means that the presence of the amino group in dcIII makes fragmentation via the loss of water energetically favored. Therefore, the dcIII spectrum also shows signals that are not registered in the carminic acid spectrum: ions at *m*/*z* 400 and 370, attributed to the primary fragmentation of the sugar ring [^0,3^X − H]^−^ and [^0,2^X − H]^−^, as well as another two at *m*/*z* 382 and 352, corresponding to the additional loss of water [^0,3^X − H − H_2_O]^−^ and [^0,2^X − H − H_2_O]^−^, respectively.

The chromatogram of cochineal extract reconstructed for *m*/*z* 475, apart from the signal from dcII, shows a peak at a retention time of 15.5 min. Only two ions are observed in its MS/MS spectrum; the first corresponds to a diagnostic neutral loss of CO_2_ (*m*/*z* 431), confirming the presence of a carboxyl group, and the second one at *m*/*z* 269 is formed upon the detachment of the whole glucose moiety. Such fragmentation is characteristic of *O*-glycosides, so this compound was identified as 6-*O*-α-D-glucopyranoside of flavokermesic acid (dcOfka).

#### Non-glycosidic colorants

Kermesic acid and flavokermesic acid differ in the presence of the hydroxyl substituent at the C-5 position, so their molecular masses differ by 16 Da. The former is an aglycone of carminic acid, and the latter an aglycone of dcII. The MS/MS spectra of the quasi-molecular ions of kermesic acid (*m*/*z* 329) and flavokermesic acid (*m*/*z* 313) show only peaks attributed to the subsequent losses of CO_2_ (44 Da) and CO (28 Da). In the spectrum of kermesic acid there are [M − H − CO_2_]^−^ at *m*/*z* 285, [M − H − CO_2_ − CO]^−^ at *m*/*z* 257, [M − H − 2CO_2_ − CO]^−^ at *m*/*z* 213, [M − H − 2CO_2_ − 2CO]^−^ at *m*/*z* 185, and [M − H − 3CO_2_ − CO]^−^ at *m*/*z* 169. The first three signals in the spectrum of flavokermesic acid have the same origin, and they are registered at *m*/*z* values that are lower by 16, i.e., 269, 241, and 197, respectively, while further fragmentation also leads to the formation of the [M − H − 2CO_2_]^−^ ion at *m*/*z* 225.

### New anthraquinone colorants (dc1–dc9) found in pre-Columbian yarns

**Fig. 2a–b:**
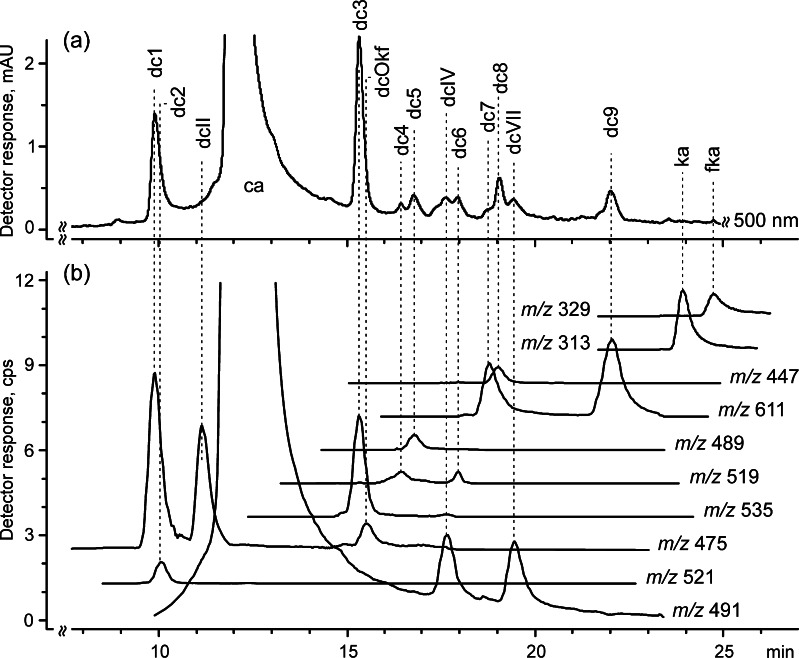
Chromatograms of the extract from fiber KPT8G (the same compounds are present in fiber 15885) registered using **a** a UV-vis detector at 500 nm, and **b** an ESI MS detector operated in negative ion mode (reconstructed for quasi-molecular ions, cf. Table 2)

All of the compounds found in American cochineal except for dcIII (containing an amino group) were also present in the extracts of both pre-Columbian threads. As well as those compounds, nine other colorants (dc1–dc9) were registered in the chromatograms (Fig. 2) and identified based on MS/MS spectra and high-resolution data. According to the best knowledge of the authors, these nine compounds have not been reported previously in the literature.

#### Carboxylic acids with the carboxyl group not involved in derivative formation

**Fig. 3a–j:**
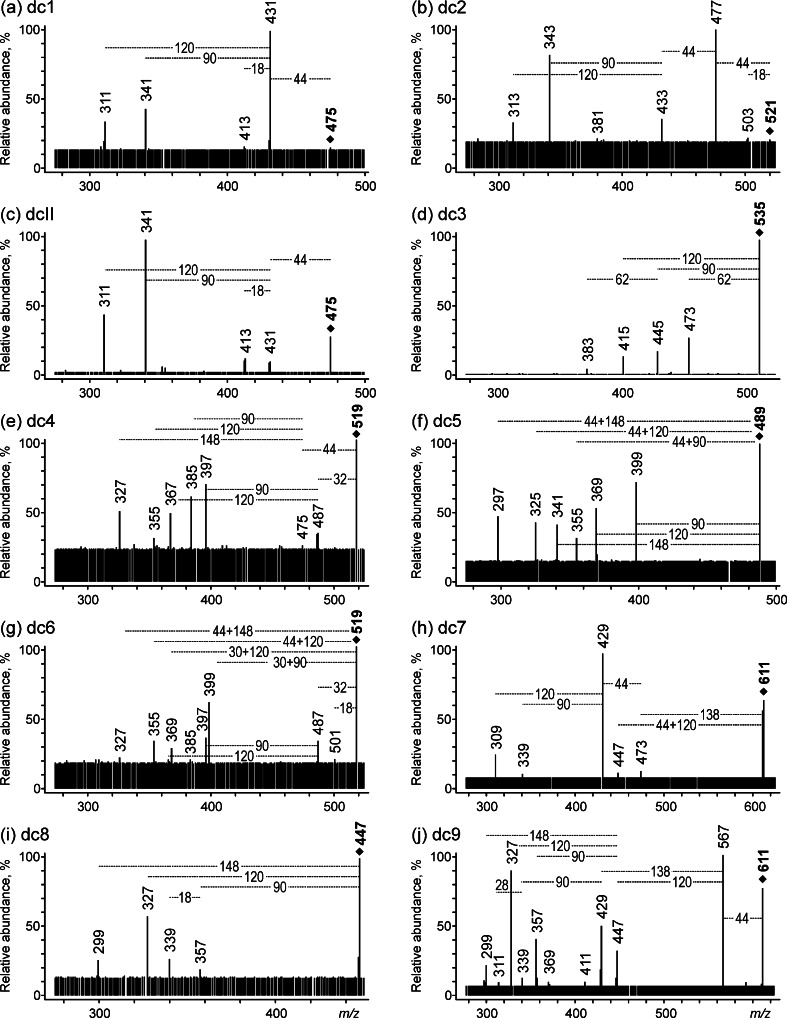
ESI QqQ MS product ion spectra (mother ions [M − H]^−^, CE 20 V) of **a** dc1, **b** dc2, **c** dcII, **d** dc3, **e** dc4, **f** dc5, **g** dc6, **h** dc7, **i** dc8, and **j** dc9

**Fig. 4:**
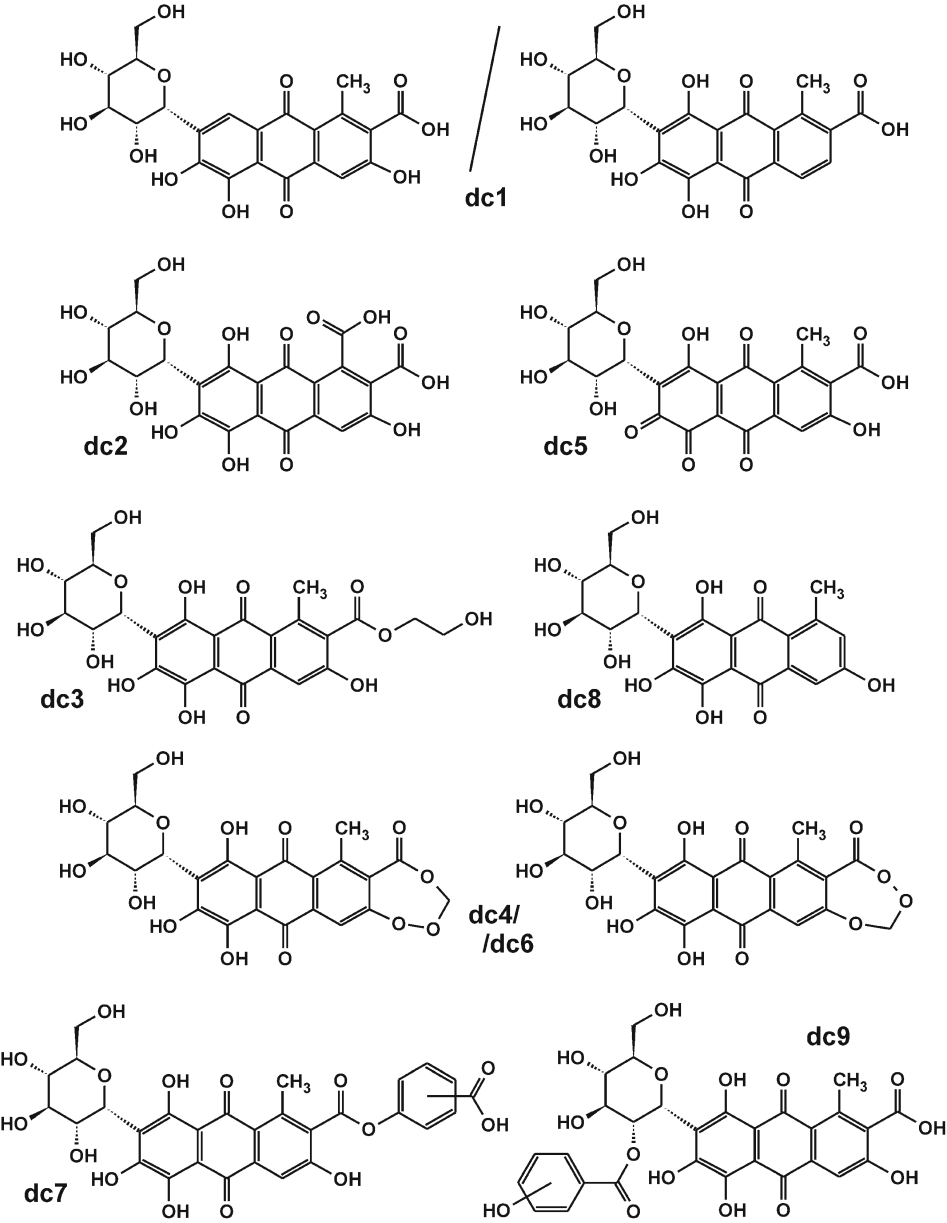
Proposed structures of carminic acid derivatives found in pre-Columbian threads

The fragmentation pattern of dc1 is identical to that of dcII, which has already been discussed (Fig. 3). The only difference is the observed signal intensities; in the case of dc1, the most intense is the [M − H − CO_2_]^−^ ion at *m*/*z* 431, while signals resulting from further cross-ring cleavages of the glycoside moiety are weaker. These two compounds must be isomers that differ in the positions of the hydroxyl groups in the aglycone (in the dcII molecule they are situated at the C3, C6, and C8 positions). Taking into account the shorter retention time of dc1 (indicating its lower hydrophobicity), two structures can be postulated for this compound, one with hydroxyl groups at C3, C5, and C6 and a second with the groups at the C5, C6, and C8 positions (Fig. 4). This hypothesis derives from an analogy to another group of compounds: alizarin, xanthopurpurin, and quinizarin, which differ from each other in the locations of the two hydroxyl groups in the anthraquinone aromatic rings [21, 22]. During separation by reversed-phase HPLC, the shortest retention time is registered for alizarin, which has two hydroxyl groups in the *ortho* position (as in dc1), while the *meta* configuration of these groups in xanthopurpurin (as in dcII) increases its hydrophobicity and prolongs elution. Among these discussed compounds, the longest retention is observed for the *para* isomer, quinizarin. Thus, the structure of dc1 with hydroxyl groups at the C3, C5, and C8 positions can be excluded.

The MS/MS spectrum of dc2 (mother ion at *m*/*z* 521) is very similar to the spectrum of carminic acid, but the signals are shifted by 14 Da to lower *m*/*z*. This suggests that dc2 is a derivative of carminic acid. The exception is the double loss of CO_2_ (ions at *m*/*z* 477 and 447). The accurate mass of its quasi-molecular ion as determined by ESI QIT/ToF MS is 521.0589 Da (diff. 3.07) and its elemental composition is C_22_H_17_O_15_, while the mass of the ion formed by the loss of CO_2_ is 477.0582 (diff. 1.68, elemental composition C_21_H_17_O_13_). These results allow us to identify dc2 as a dicarboxylic derivative of carminic acid in which the methyl group at the C1 position is replaced by a carboxylic group.

In the MS/MS spectrum of dc5 ([M–H]^−^, *m*/*z* 489), the main signals are observed at *m*/*z* 399 [^0,3^X − H]^−^, *m*/*z* 369 [^0,2^X − H]^−^, and *m*/*z* 341 [^0,1^X − H]^−^ (losses of 90, 120, and 148 Da, which are typical of *C*-glucosides). There is no signal from the [M − H − CO_2_]^−^ ion, but the ions [^0,3^X − H − CO_2_]^−^ at *m*/*z* 355, [^0,2^X − H − CO_2_]^−^ at *m*/*z* 325, and [^0,1^X − H − CO_2_]^−^ at *m*/*z* 297 are present instead, indicating that fragmentation of the sugar ring is energetically favored over the loss of CO_2_. Moreover, a quasi-molecular ion of dc5 with *m*/*z* 489 (2 Da less than the mass of carminic acid) suggests that this compound is also a derivative of carminic acid and contains a methyl group instead of a hydroxyl group or a group with two hydrogen atoms less (an additional unsaturated bond). The second hypothesis was confirmed by an accurate mass spectrum registered via ESI QIT/ToF MS in which the peak of [M–H]^−^ is situated at *m*/*z* 489.0677 (elemental composition of C_22_H_17_O_13_). Based on these results which, together with the product ions, indicate that the unsaturated bond is located in the aglycone, dc5 was identified as a dehydrocarminic acid formed by the oxidative dehydrogenation of carminic acid.

The accurate mass of the [M − H]^−^ quasi-molecular ion of dc9 is 611.1075 (elemental composition of C_29_H_23_O_15_). The difference (C_7_H_4_O_2_) between the elemental composition of carminic acid and that of dc9 can be attributed to a benzoate moiety. In order to characterize the bond formed between the substituent and carminic acid as well as its position in the host molecule, the MS/MS spectrum of the quasi-molecular ion serving as the mother ion was carefully analyzed. Its fragmentation pattern was very rich and consisted of many signals typical of glycosidic derivatives. The presence of an intense signal at *m*/*z* 567 corresponding to the loss of CO_2_ proves that the carboxyl group of carminic acid is not involved in this bond formation. Signals at *m*/*z* 429 (obs. 429.0852, diff. for C_21_H_18_O_10_ 5.83) and 447 (obs. 447.0971, diff. for C_21_H_20_O_11_ 8.50) correspond to the ions formed upon the loss of hydroxybenzoic acid (138 Da, C_7_H_6_O_3_, HA) and an analogous ketene molecule (120 Da, C_7_H_4_O_2_, Ket), respectively. Such fragmentation is well known [23, 24] and explained by the decomposition of acylated glycosides due to the dissociation of an ester bond (Ket) or a neighboring bond between the oxygen atom and pyranose ring (HA). Moreover, the possibility of ketene loss (oxomethylene cyclohexadienone, O=C_6_H_4_=C=O) proves that a hydroxyl group in hydroxybenzoic acid is situated in the *ortho* or *para* position, while the presence of ions [^0,3^X − H − CO_2_ − HA]^−^ at *m*/*z* 339 (obs. 339.0496, diff. for C_18_H_11_O_7_ 4.13) and [^0,3^X − H − CO_2_ − HA − CO]^−^ at *m*/*z* 311 (obs. 311.0551, diff. for C_17_H_11_O_6_ 3.21) is very significant, as the loss of CO is possible only when hydroxybenzoic acid is esterified via a C-2′-hydroxyl group of the dc9 molecule.

#### Derivatives of carminic acid with the carboxyl group involved in the formation of identified species

In the MS/MS spectrum of dc3, losses of 90 (*m*/*z* 445) and 120 Da (*m*/*z* 415) are registered, which are characteristic of *C*-glucosides. However, the most intense signal, which occurs at *m*/*z* 473 and corresponds to a loss of 62 Da and a lack of 44-Da detachment, indicates that a substituent is attached to the carboxyl group. In the accurate QIT/ToF MS/MS spectrum of the quasi-molecular ion (obs. *m*/*z* 535.1092; elemental composition C_24_H_23_O_14_, which differs from the elemental composition of carminic acid by a C_2_H_4_O unit) there is a peak at *m*/*z* 473.0724 (diff. for C_22_H_17_O_12_ 0.21 ppm) that can be attributed to the loss of a C_2_H_6_O_2_ fragment. This unique signal allows us to identify the examined compound as the ethylene glycol ester of carminic acid.

The MS/MS spectra of dc4 and dc6 ([M–H]^−^, *m*/*z* 519) show ions formed upon the loss of CO_2_, as well as a series of ions that are characteristic of *C*-glucosides (losses of 90, 120, and 148 Da, i.e., C_3_H_6_O_3_, C_4_H_8_O_4_, and C_5_H_8_O_5_, from the glucose ring, but accompanied here by losses of other small molecules): at *m*/*z* 475 ([M − H − CO_2_]^−^), *m*/*z* 385 ([^0,3^X − H − CO_2_]^−^), *m*/*z* 355 ([^0,2^X − H − CO_2_]^−^), *m*/*z* 327 ([^0,1^X − H − CO_2_]^−^), *m*/*z* 397 ([^0,3^X − H − O_2_]^−^), *m*/*z* 367 ([^0,2^X − H − O_2_]^−^), *m*/*z* 339 ([^0,1^X − H − O_2_]^−^), *m*/*z* 399 ([^0,3^X − H − CH_2_O]^−^), and *m*/*z* 369 ([^0,2^X − H − CH_2_O]^−^).

In the ESI QIT/ToF MS spectra, other decomposed radical ions with accurate masses of 312.0297 and 284.0319 were observed. These ions are formed by the homolytic detachment of a whole glucose moiety (Glc) accompanied by carbonyl group fragmentation. The elemental compositions of these ions are C_16_H_8_O_7_^•^ (diff. 6.73 ppm) and C_15_H_8_O_6_^•^ (diff. 2.46 ppm), respectively, and they differ in a CO unit. The registration of such ions proves that substituents at positions C-1, C-3, C-5, C-6, and C-8 are not involved in their formation (the ion at *m*/*z* 284 perfectly reflects the structure of the ion obtained by heterolytic cleavage of the bond between the glucose moiety and the aglycone of carminic acid, cf. Scheme 1).

Accurate masses of the quasi-molecular ions of dc4 and dc6 are 519.0778 Da and 519.0779 Da, respectively (elemental composition of C_23_H_19_O_14_, corresponding to the composition of carminic acid enlarged by a CO unit). Ions formed upon the loss of a 32-Da fragment (*m*/*z* 487) are observed in the ESI QqQ MS/MS mass spectra of both quasi-molecular ions, which may suggest detachment of a methanol molecule. Signals at m/z 487 are not observed in high resolution ESI QIT/ToF MS spectra, but the above hypothesis is confirmed by the presence of another pairs of signals registered in spectra of dc4 and dc6: at m/z 399.0395 and 367.0434 (elemental compositions: C19H11O10, diff. 9.27 ppm, and C19H11O8, diff. 6.81 ppm, respectively). These results clearly indicate that the 32-Da loss observed in this case does not correspond to detachment of CH_3_OH but detachment of an O_2_ molecule. Such a loss has already been reported for hydroperoxides in both positive [25, 26] and negative [27, 28] ion modes. Hence, it is proposed that dc4 and dc6 contain a peroxy group (−O−O−).

The lack of signals indicating the presence of a methoxy or ester group in the structures of the examined isomeric compounds dc4 and dc6 suggests that they contain a peroxy group, which probably forms through the creation of a direct bond between CO and the oxygen atom from the carboxyl group or the adjacent hydroxyl group; their structures probably differ only in the construction of the peroxy group (Fig. 4).

**Scheme 2:**
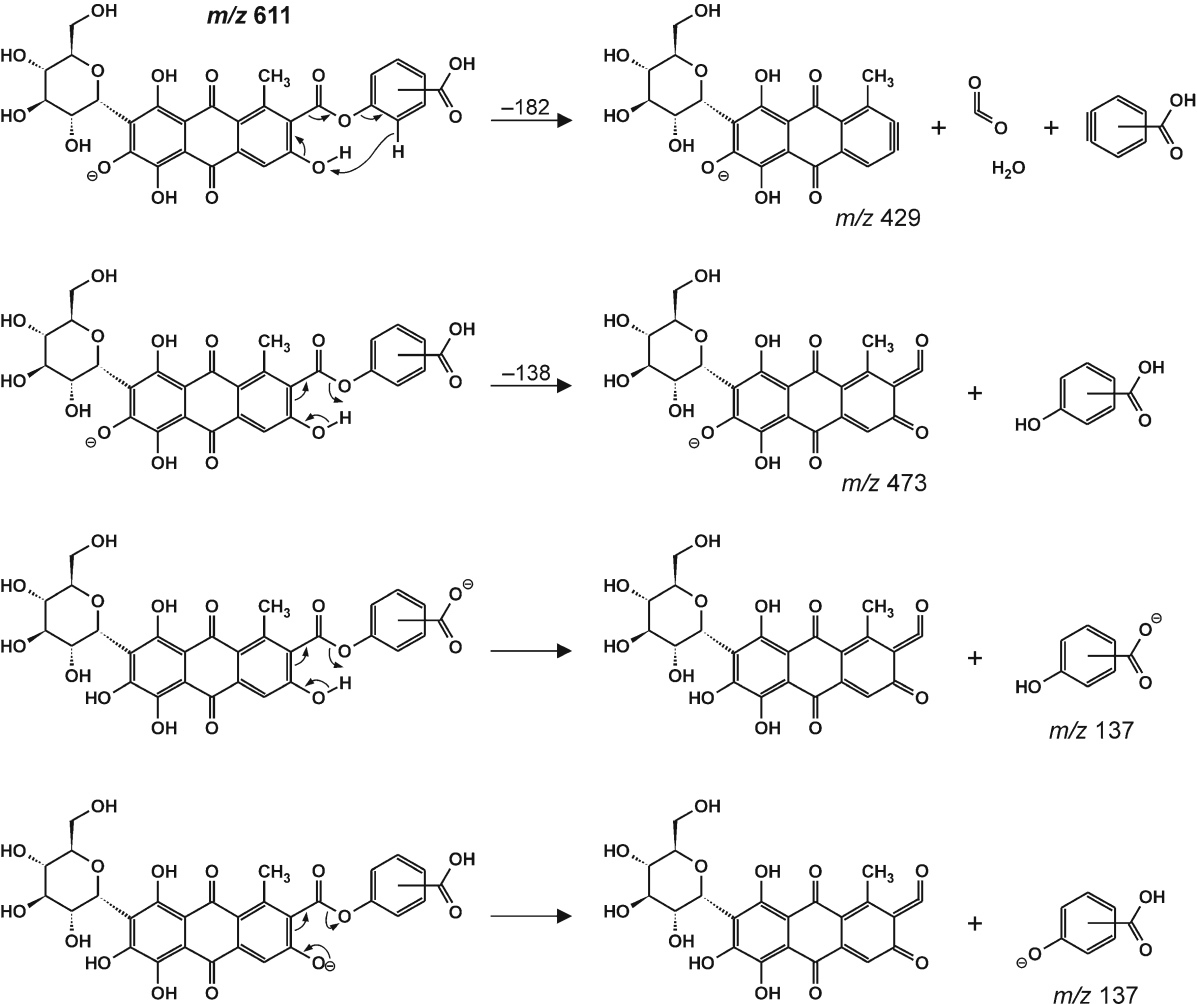
Proposed mechanism for the fragmentation of the *m*/*z* 611 ion from dc7

The quasi-molecular [M − H]^−^ ion of dc7 is registered at *m*/*z* 611 (611.1037 in the high-resolution mass spectrum), corresponding to an elemental composition of C_29_H_23_O_15_, identical to the composition of dc9. It can therefore be identified as an isomer of dc9, but these molecules show different MS/MS spectra and that obtained for dc7 is substantially poorer. In it, only two relatively intense signals at *m*/*z* 429 and 309 are observed. The absence of a peak corresponding to the loss of a 44-Da fragment, as well as a difference of C_7_H_4_O_2_ (a benzoate group) between the elemental compositions of carminic acid and dc7, indicate that the carboxyl group is esterified by hydroxybenzoic acid. A similar phenomenon—a lack of detachment of CO_2_ from the hydroxybenzoic carboxylic group (not engaged in bonding)—has already been reported for diesters of aliphatic dicarboxylic acids with hydroxybenzoic acid [29]. Assuming such a structure for dc7, the formation of ions at *m*/*z* 429 (obs. *m*/*z* 429.0809, diff. for C_21_H_18_O_10_ 4.19) can be explained by atypical detachment of the carbophenoxy ester group together with the neighboring hydroxyl group (182 Da, C_8_H_6_O_5_), which probably originates from a charge-remote mechanism based on the elimination of CO_2_, H_2_O, and the hydroxybenzoic acid moiety (Scheme 2).

The signal at *m*/*z* 309 corresponds to the 120-Da loss of a glucose moiety from the ion at *m*/*z* 429. Ions registered at *m*/*z* 473 are probably formed via the same mechanism, as discussed above for the ion at *m*/z 429 but with the detachment of hydroxybenzoic acid and the formation of ketene and carbonyl groups at the C-2 and C-3 positions, respectively. This mechanism is also responsible for the formation of the ion at *m*/*z* 137 corresponding to deprotonated hydroxybenzoic acid, as confirmed by the presence of the signal at *m*/*z* 93 ([137 − CO_2_]^−^).

The last small signal in the spectrum, at *m*/*z* 447, can be attributed to the ion formed by the detachment of the entire ester group. High-resolution measurements (obs. *m*/*z* 447.0910, diff. for C_21_H_19_O_11_ 5.14) allowed us to confirm that this 164 Da loss was not caused by glucose detachment.

#### Non-carboxylic compound

The dc8 peak corresponding to the decarboxylated ion was not observed in the MS/MS spectrum. However, except for this, its fragmentation pattern is almost the same as that registered for carminic acid: there are signals at *m*/*z* 357 [^0,3^X − H]^−^, *m*/*z* 327 [^0,2^X − H]^−^, and *m*/*z* 299 [^0,1^X − H]^−^. Based on these results, which were confirmed by high-resolution QIT/ToF MS measurements, dc8 was identified as decarboxylated carminic acid.

## Conclusions

The coupling of reversed-phase HPLC with UV-vis and ESI MS/MS detection was shown to be a particularly effective tool for analyzing animal colorants in historical samples dyed with American cochineal. It allows the detection and even standardless identification of unknown compounds present at trace levels in 0.2–0.3 mg samples. This system was found to be especially useful for examining a variety of derivatives of the main colorant, carminic acid. Even only small differences in the *m*/*z* values of quasi-molecular ions are the basis for characterizing tiny differences in the structures of its derivatives. Characteristic signals obtained after the fragmentation of the compounds provide additional information on the lost neutrals, and thus on the functional groups and substituents that are the sources of these losses (Table 3). Nevertheless, sometimes only data obtained using a high-resolution detector, e.g., ToF, will allow the correct recognizition of recorded signals, for instance the observation of O_2_ loss, which is characteristics of peroxide fragmentation.

**Table 3:** Processes observed during the fragmentation of derivatives of carminic acid and the resulting changes in mass

Transformation	Nominal mass shift, ΔDa	Exact mass shift, ΔDa	Exact mass shift, mDa	Formula change
Null	0	0	0	Carminic acid
Deglucosylation + dehydroxylation	−178	−178.0477	−47.7	−C_6_H_10_O_5_ − O
Deglucosylation	−162	−162.0528	−52.8	−C_6_H_10_O_5_
Decarboxylation	−44	−43.9898	−10.2	−CO_2_
Dehydroxylation	−16	−15.9949	−5.1	−O
Oxidation	−2	−2.0157	−15.6	−H_2_
Dehydroxylation + amination	−1	−0.9840	+1984.0	−O + NH
Amination	+15	+15.0109	−10.9	+NH
Methyleneoxy conjugation	+28	+27.9949	+5.1	+CO
Demethylation + carboxylation	+30	+29.9747	−25.3	−CH_2_ + CO_2_
Hydroxyethyl conjugation	+44	+44.0262	−26.2	+C_2_H_4_O
Hydroxybenzoic acid conjugation	+120	+120.0211	−21.1	+C_7_H_4_O_2_

The use of the discussed instrumental setup allowed us to identify the pattern of colorants present in pre-Columbian threads dyed with American cochineal, which could probably serve as a fingerprint of the textiles that were produced in the Peruvian region.

## References

[1] Cardon D (2007) Natural dyes: sources, tradition, technology, science. Archetype, London

[2] Phipps E (2010). Cochineal red: the art history of a color.

[3] Hofenk de Graaff JH (2004) The colorful past. Origins, chemistry and identification of natural dyestuffs. Archetype, London

[4] Lech K, Połeć-Pawlak K, Jarosz M (2008). Mass spectrometry in identification of color components of natural organic dyestuffs used in art. Chem Anal.

[5] Rosenberg E (2008). Characterisation of historical organic dyestuffs by liquid chromatography–mass spectrometry. Anal Bioanal Chem.

[6] Domon B, Costello CE (1988). A systematic nomenclature for carbohydrate fragmentations in FAB-MS/MS spectra of glycoconjugates. Glycoconj J.

[7] Cuyckens F, Claeys M (2004). Mass spectrometry in the structural analysis of flavonoids. J Mass Spectrom.

[8] Jin W, Wang YF, Ge RL, Shi HM, Jia CQ, Tu PF (2007). Simultaneous analysis of multiple bioactive constituents in *Rheum tanguticum* Maxim. ex Balf. by high-performance liquid chromatography coupled to tandem mass spectrometry. Rapid Commun Mass Spectrom.

[9] Lech K, Jarosz M (2011). Novel methodology for the extraction and identification of natural dyestuffs in historical textiles by HPLC–UV-Vis–ESI MS. Case study: chasubles from the Wawel Cathedral collection. Anal Bioanal Chem.

[10] Qiu X, Zhang J, Huang Z, Zhu D, Xu W (2013). Profiling of phenolic constituents in *Polygonum multiflorum* Thunb. by combination of ultra-high-pressure liquid chromatography with linear ion trap-Orbitrap mass spectrometry. J Chromatogr A.

[11] Wouters J, Verhecken A (1987). The chemical nature of flavokermesic acid. Tetrahedron Lett.

[12] Peggie DA, Hulme AN, McNab H, Quye A (2008) Towards the identification of characteristic minor components from textiles dyed with weld (*Reseda luteola* L.) and those dyed with Mexican cochineal (*Dactylopius coccus* Costa). Microchim Acta 162:371–380

[13] Wouters J, Rosario-Chirinos N (1992) Dye analysis of pre-Columbian Peruvian textiles with high-performance liquid chromatography and diode-array detection. J Am Inst Conserv 31:237–255

[14] Zhang X, Boytner R, Cabrera JL, Laursen R (2007). Identification of yellow dye types in pre-Columbian Andean textiles. Anal Chem.

[15] Serrano A, Sousa MM, Hallett J, Lopes JA, Oliveira C (2011). Analysis of natural red dyes (cochineal) in textiles of historical importance using HPLC and multivariate data analysis. Anal Bioanal Chem.

[16] Lech K, Jarosz M (2014) In: Zuo Y (ed) High-performance liquid chromatography (HPLC): principles, practices and procedures. Nova Science Publishers Inc., New York

[17] Stathopoulou K, Valianou L, Skaltsounis AL, Karapanagiotis I, Magiatis P (2013). Structure elucidation and chromatographic identification of anthraquinone components of cochineal (*Dactylopius coccus*) detected in historical objects. Anal Chim Acta.

[18] Karapanagiotis I, Mantzouris D, Kamaterou P, Lampakis D, Panayiotou C (2011). Identification of materials in post-Byzantine textiles from Mount Athos. J Archaeol Sci.

[19] Mantzouris D, Karapanagiotis I, Valianou L, Panayiotou C (2011). HPLC–DAD–MS analysis of dyes identified in textiles from Mount Athos. Anal Bioanal Chem.

[20] Wu LZ, Zhang XP, Xu XD, Zheng QX, Yang JS, Ding WL (2013) Characterization of aromatic glycosides in the extracts of *Trollius* species by ultra high-performance liquid chromatography coupled with electrospray ionization quadrupole time-of-flight tandem mass spectrometry. J Pharm Biomed Anal 75:55–63 10.1016/j.jpba.2012.11.015 23312385

[21] Surowiec I, Szostek B, Trojanowicz M (2007). HPLC-MS of anthraquinoids, flavonoids, and their degradation products in analysis of natural dyes in archeological objects. J Sep Sci.

[22] Rafaëlly L, Héron S, Nowik W, Tchapla A (2008). Optimisation of ESI-MS detection for the HPLC of anthraquinone dyes. Dyes Pigments.

[23] Gu D, Yang Y, Bakri M, Chen Q, Xin X, Aisa HA (2013). A LC/QTOF-MS/MS application to investigate chemical compositions in a fraction with protein tyrosine phosphatase 1B inhibitory activity from *Rosa rugosa* flowers. Phytochem Anal.

[24] Kilár A, Dörnyei Á, Kocsis B (2013). Structural characterization of bacterial lipopolysaccharides with mass spectrometry and on- and off-line separation techniques. Mass Spectrom Rev.

[25] Reis A, Domingues P, Ferrer-Correia AJV, Domingues MRM (2004). Tandem mass spectrometry of intact oxidation products of diacylphosphatidylcholines: evidence for the occurrence of the oxidation of the phosphocholine head and differentiation of isomers. J Mass Spectrom.

[26] Tudella J, Nunes FM, Paradela R, Evtuguin DV, Domingues P, Amado F, Coimbra MA, Barros AI, Domingues MR (2011). Oxidation of mannosyl oligosaccharides by hydroxyl radicals as assessed by electrospray mass spectrometry. Carbohydr Res.

[27] Domingues P, Simoes MMQ, Cardoso AM, Cavaleiro AMV, Cavaleiro JAS, Johnstone RAW, Ferrer-Correia AJ (1999). Negative chemical ionisation and collision induced fragmentations of deprotonated hydroperoxides. Rapid Commun Mass Spectrom.

[28] Maciel E, Domingues P, Marques D, Simões C, Reis A, Oliveira MM, Videira RA, Peixoto F, Domingues MRM (2011) Cardiolipin and oxidative stress: identification of new short chain oxidation products of cardiolipin in in vitro analysis and in nephrotoxic drug-induced disturbances in rat kidney tissue. Int J Mass Spectrom 301:62–73

[29] Nishshanka U, Attygalle AB (2008). Low-energy collision-induced fragmentation of negative ions derived from diesters of aliphatic dicarboxylic acids made with hydroxybenzoic acids. J Mass Spectrom.

